# Cervical lymph node metastases in thoracic esophageal cancer: a systematic review

**DOI:** 10.1093/dote/doag068

**Published:** 2026-07-06

**Authors:** M E Sanders, F E Lammes, S Kalash, S van der Horst, S Mook, T J Weijs, J E Freund, B L A W Weusten, G J Meijer, N Haj Mohammad, A Zeyara, J P Ruurda, R van Hillegersberg

**Affiliations:** Department of Surgery, University Medical Center Utrecht, Utrecht, The Netherlands; Department of Radiation Oncology, University Medical Center Utrecht, Utrecht, The Netherlands; Department of Surgery, University Medical Center Utrecht, Utrecht, The Netherlands; Department of Surgery, University Medical Center Utrecht, Utrecht, The Netherlands; Department of Surgery, University Medical Center Utrecht, Utrecht, The Netherlands; Department of Radiation Oncology, University Medical Center Utrecht, Utrecht, The Netherlands; Department of Surgery, University Medical Center Utrecht, Utrecht, The Netherlands; Department of Pathology, University Medical Center Utrecht, Utrecht, The Netherlands; Department of Gastroenterology and Hepatology, University Medical Center Utrecht, Utrecht, The Netherlands; Department of Radiation Oncology, University Medical Center Utrecht, Utrecht, The Netherlands; Department of Medical Oncology, University Medical Center Utrecht, Utrecht, The Netherlands; Department of Surgery, University Medical Center Utrecht, Utrecht, The Netherlands; Department of Surgery, Skåne University Hospital, Lund, Sweden; Department of Surgery, University Medical Center Utrecht, Utrecht, The Netherlands; Department of Surgery, University Medical Center Utrecht, Utrecht, The Netherlands

**Keywords:** cervical lymph node metastases, esophageal carcinoma, lymphatic spread, staging classification, three-field lymphadenectomy

## Abstract

Cervical lymph node metastases in thoracic esophageal cancer represent a controversial pattern of nodal spread, with inconsistent classification across staging systems and substantial variation in reported management and outcomes. Although frequently categorized as distant metastatic disease, their location within the longitudinal lymphatic drainage pathways of the esophagus raises uncertainty regarding their biological and clinical impact. A systematic review was conducted. PubMed, Embase, and the Cochrane Library were searched for studies reporting treatment strategies and survival outcomes in patients with thoracic esophageal cancer (squamous cell carcinoma or adenocarcinoma) presenting with cervical and/or supraclavicular lymph node metastases at initial staging. The primary outcome was overall survival. In total, 19 studies comprising 1669 patients were included, predominantly retrospective in design. Most cohorts consisted primarily of squamous cell carcinoma, with limited representation of adenocarcinoma. Definitions and classification of cervical nodal disease varied widely, with inconsistent anatomical stratification and limited reporting of disease extent and nodal burden. Survival outcomes differed substantially. In selected patients treated with curative intent, 3-year overall survival reached up to 72%, and 5-year overall survival up to 41%. Favorable outcomes were most often observed in anatomically defined and carefully selected cohorts. Cervical lymph node metastases in thoracic esophageal cancer should not be regarded as a uniform disease entity. Available evidence suggests that involvement confined to the longitudinal paraesophageal and parajugular drainage pathway may behave more similarly to locoregional (N) disease than to unequivocal M1 systemic metastatic spread. In selected patients treated with curative intent, including neoadjuvant therapy followed by esophagectomy with three-field lymphadenectomy, long-term survival has been reported. Anatomically precise stratification and consistent reporting are essential to improve prognostic interpretation and guide treatment decisions.

## Graphical Abstract

**Graphical Abstract:**
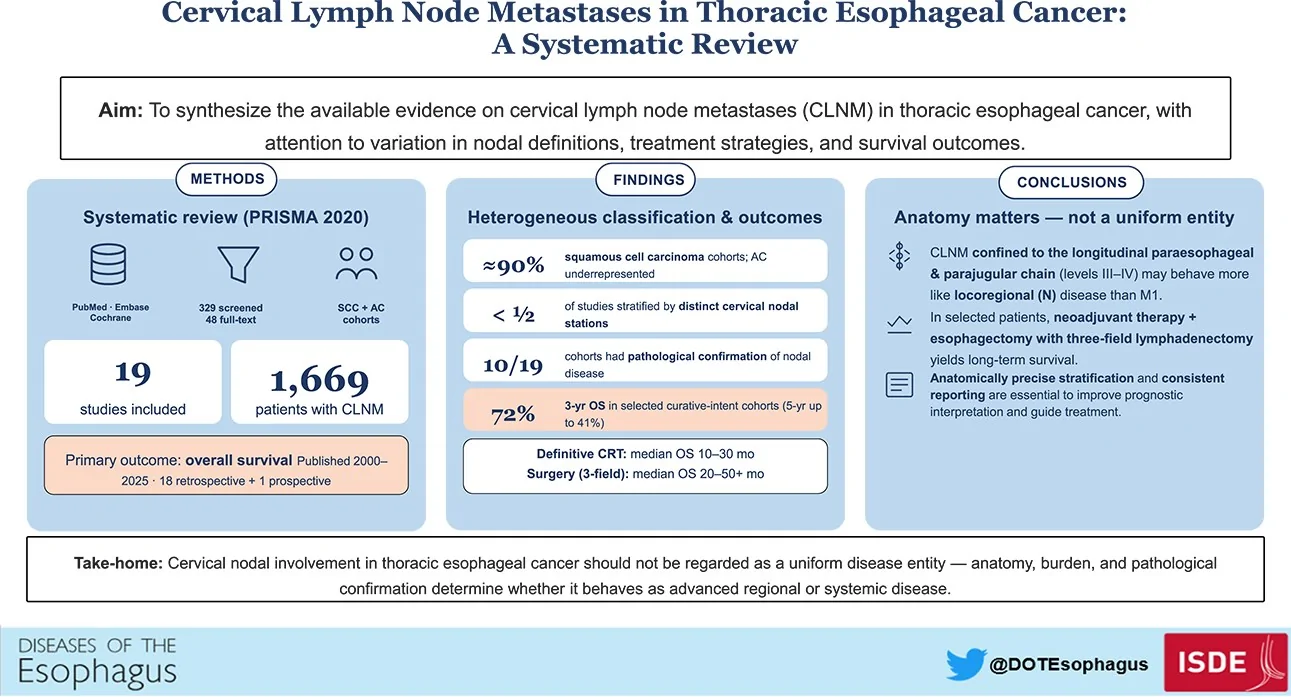

## INTRODUCTION

Esophageal cancer is a major global health problem with poor long-term survival. Prognosis is strongly influenced by early lymphatic dissemination. Lymph node involvement represents one of the most important determinants of both survival and treatment strategy across histological subtypes. While squamous cell carcinoma (SCC) remains the predominant subtype worldwide, the incidence of adenocarcinoma (AC) has increased substantially in Western countries over recent decades.^1^^,^^2^ Much of the available evidence regarding advanced nodal disease is derived from SCC, with comparatively limited data in AC, which differs in pathophysiology, epidemiology, and treatment response.^3^

Cervical lymph node metastases (CLNM) represent a particularly controversial pattern of nodal spread in thoracic esophageal cancer. The esophagus is characterized by an extensive longitudinal lymphatic network, enabling cranial dissemination to cervical lymph node stations without obligatory involvement of thoracic or mediastinal nodes.^4^ This anatomical feature raises uncertainty as to whether cervical nodal involvement reflects locoregional extension or true systemic metastatic spread. In many Western treatment paradigms, however, the presence of CLNM has historically been interpreted as distant metastatic disease, frequently resulting in non-curative treatment approaches.

Differences between staging systems further reflect this uncertainty. In the American Joint Committee on Cancer (AJCC)/Union for International Cancer Control (UICC) TNM classification, regional lymph nodes are defined as those within the esophageal lymphatic drainage areas, including paraesophageal lymph nodes along the entire length of the esophagus, while excluding supraclavicular lymph nodes.^5^ In contrast, the Japanese Esophageal Society (JES) Classification applies a broader anatomical definition and considers cervical lymph node involvement, including supraclavicular nodes, as regional disease, particularly for upper and middle thoracic tumors.^6–8^ In addition, CLNM and supraclavicular lymph node metastases (SCLNM) are frequently used interchangeably in the literature, despite referring to anatomically distinct nodal regions, contributing to heterogeneity in patient classification and outcome reporting.

Esophageal cancer staging has previously evolved in response to similar controversies. Notably, celiac axis lymph node metastases were historically classified as distant disease (M1a) but from the AJCC TNM 7^th^ edition on were classified as regional lymph nodes based on anatomical and outcome data.^9^ Such precedents illustrate that staging frameworks may require refinement when anatomical pathways and survival patterns suggest a more nuanced biological behavior.

Treatment strategies mirror these divergent conceptual frameworks. In many Western centers, curative treatment for non-metastatic thoracic esophageal cancer typically consists of neoadjuvant therapy followed by esophagectomy with two-field lymphadenectomy, whereas patients presenting with CLNM are often managed with definitive chemoradiotherapy or palliative-intent.^10^ In contrast, surgical practice in Japan has historically favored three-field lymphadenectomy, including cervical lymph node dissection, with retrospective series reporting long-term survival outcomes in selected patients comparable to those observed in patients with other regional nodal disease.^11–13^

Collectively, these observations challenge a binary classification of cervical nodal involvement as either purely locoregional or unequivocally metastatic disease. Conceptually, cervical nodal metastases may occupy an intermediate position between regional and systemic spread, depending on the anatomic location.

The aim of this systematic review is to synthesize the available evidence on CLNM in thoracic esophageal cancer, with particular attention to variation in nodal definitions, treatment strategies, and survival outcomes. By integrating data across different staging frameworks and therapeutic approaches, this review seeks to clarify how cervical nodal involvement has been conceptualized and managed, and to identify factors that may influence interpretation of prognosis and treatment effect.

## METHODS

### Registration and reporting standards

This systematic review was prospectively registered in the PROSPERO database (CRD42024548058). The review was conducted and reported in accordance with the Preferred Reporting Items for Systematic Reviews and Meta-Analyses (PRISMA) 2020 guidelines.

### Search strategy

A systematic literature search was performed in PubMed, Embase, and the Cochrane Library. The search strategy combined controlled vocabulary terms (Medical Subject Headings [MeSH] and Emtree terms, where applicable) with free-text terms related to esophageal cancer and cervical and/or supraclavicular lymph node metastases. The full search strategy for each database is provided in Supplementary Tables S1-S3. No restrictions on publication year were applied. The search was last updated on 20 October 2025.

### Eligibility criteria and study selection

Studies were eligible for inclusion if they reported on patients with thoracic esophageal or gastroesophageal junction cancer (SCC or AC) presenting with cervical and/or supraclavicular lymph node metastases at initial staging and provided data on treatment strategies and survival outcomes. Randomized controlled trials, prospective cohort studies, and retrospective cohort studies were considered eligible. Cervical and supraclavicular nodal stations were evaluated jointly, as they are variably defined and classified across staging systems and are frequently reported together in clinical studies.

Studies were excluded if they were case reports, small case series (fewer than 10 patients), conference abstracts, editorials, review articles, or preclinical laboratory studies. The search was limited to articles published in English.

After removal of duplicates, titles and abstracts were screened independently by two reviewers (MS and SK), followed by full-text evaluation of potentially eligible articles. Disagreements were resolved through discussion and consensus.

### Data extraction and outcomes

Data extraction was performed using a predefined standardized form by one reviewer and independently verified by a second reviewer. Extracted variables included first author, year of publication, country of origin, study design, sample size, histological tumor type, primary tumor location, definition of CLNM, treatment strategy, duration of follow-up, and reported outcomes. Missing or unclear data were recorded as ‘not reported.’

The primary outcome of interest was overall survival. Secondary clinical outcomes included progression-free survival, disease-free survival, recurrence patterns, and treatment-related complications when reported.

In addition, given the conceptual focus of this review, we systematically extracted and analyzed data regarding the definition and classification of CLNM, including terminology used (e.g., cervical versus supraclavicular), staging labels applied (regional versus distant), classification systems referenced, anatomical nodal detail provided, and reporting of disease extent and nodal burden. These parameters were evaluated to assess heterogeneity in patient classification and its potential impact on reported outcomes.

Where multiple publications originated from overlapping institutional cohorts, studies were included only if they addressed distinct research questions, and duplicate outcome data were excluded.

### Risk of bias assessment

Methodological quality of non-randomized studies was assessed using the Newcastle–Ottawa Scale (NOS). Two reviewers independently evaluated studies across the domains of selection, comparability, and outcome assessment. Operationalization of the NOS criteria is detailed in Supplementary Table S4, and item-level scoring for each included study is presented in Supplementary Table S5. Discrepancies were resolved by consensus. Risk of bias assessments were reported descriptively and were not used as exclusion criteria.

## RESULTS

### Study selection and study characteristics

**Fig. 1:**
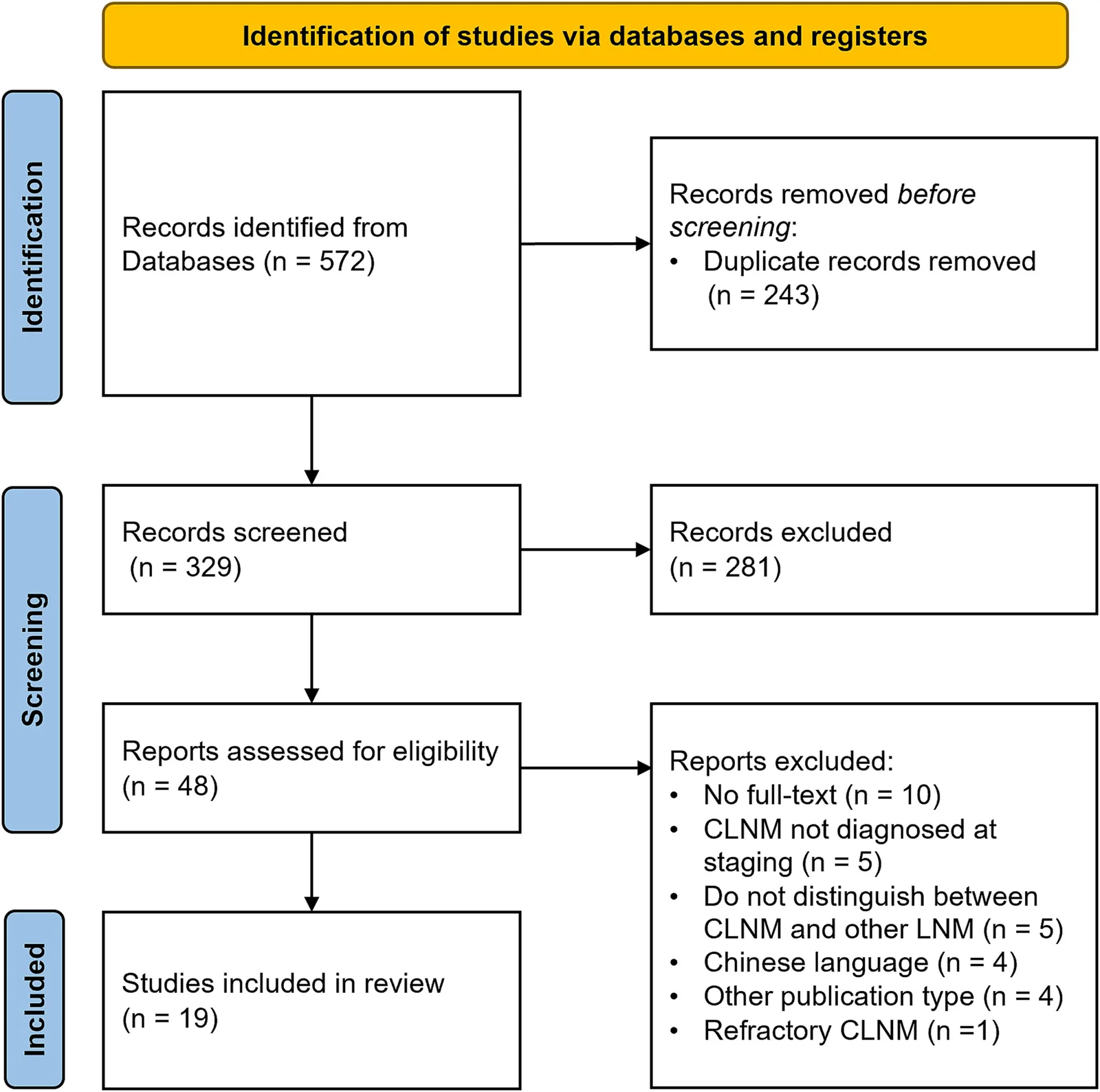
Study selection. CLNM: cervical lymph node metastases, LNM: lymph node metastases.

Following removal of duplicates, 329 records were screened by title and abstract, of which 48 full-text articles were assessed for eligibility. Nineteen studies met the predefined inclusion criteria and were included in the final analysis (Fig. 1). The included studies comprised 18 retrospective cohort studies and one prospective non-randomized study, published between 2000 and 2025.^11^^,^^13–30^

In total, 1669 patients with thoracic esophageal cancer and CLNM were included. Most studies originated from East Asia (Japan, China, Korea, and Taiwan), with three studies from Europe or the United States. SCC predominated across included studies, with 14 studies including exclusively SCC and three additional studies reporting >90% squamous histology. Only two studies included predominantly AC populations, both based on Western registry or institutional cohorts (Table 1). The majority of studies were single-center retrospective series, with considerable variation in study periods, patient selection, and treatment strategies. Primary tumor location varied across studies, with most including mixed thoracic tumors and a subset stratifying outcomes by upper, middle, or lower thoracic location. Two studies originated from overlapping institutional cohorts but addressed distinct research questions and were therefore included.^17^^,^^18^

**Table 1:** Study characteristics

Study, year	Country	Inclusion			N	Primary tumor location	Histology	
		Type	Center	Period			SCC (%)	AC (%)
Kato, 2000	Japan	RNR	Single	1990–2000	46	UT, MT, LT	>95	<5
Miyata, 2015	Japan	RNR	Single	2000–2011	57	UT, MT, LT	100	0
Liming, 2017	China	RNR	Single	2006–2013	50	UT, MT	100	0
Honma, 2017	Japan	RNR	Single	2001–2012	102	Thoracic	100	0
Kang, 2017	Korea	RNR	Single	2002–2016	34	Thoracic	100	0
Chen, 2018	Taiwan	RNR	Single	2000–2015	70	UT, MT, LT	100	0
Chen, 2018	Taiwan	RNR	Single	2000–2015	105	Thoracic	100	0
Chung, 2019	Korea	RNR	Multi	2006–2017	31	Thoracic	100	0
Sato, 2021	Japan	RNR	Single	2008–2018	18	UT, MT	100	0
Pape, 2022	Netherlands	RNR	Multi	2015–2018	118	Thoracic	100	0
Yu, 2022	China	RNR	Single	2008–2018	231	UT, MT, LT	100	0
Horst, 2023	Netherlands	PNR	Single	2015–2021	20	UT, MT, LT	35	65
Chang, 2023	USA	RNR	Multi	2010–2015	120	Thoracic	19	73
Igaue, 2024	Japan	RNR	Single	2017–2021	80	Thoracic	90	10
Park, 2024	Korea	RNR	Single	1994–2018	224	Thoracic	100	0
Li, 2024	China	RNR	Single	2010–2017	122	Thoracic	100	0
Shiraishi, 2024	Japan	RNR	Single	2003–2020	66	Thoracic	93	5
Zeng, 2024	China	RNR	Single	2019–2021	230	Cervical, UT	100	0
Zeng, 2025	China	RNR	Single	2020–2024	15	UT, MT, LT	100	0

AC, adenocarcinoma; LT, lower thoracic esophagus; MT, middle thoracic esophagus; PNR, prospective non-randomized trial; RNR, retrospective non-randomized trial; SCC, squamous cell carcinoma; UT, upper thoracic esophagus.

Methodological quality was assessed using the NOS (Supplementary Table S5). Most included studies were retrospective cohort designs with moderate methodological quality. Common limitations included lack of control for confounding, limited comparability between treatment groups, and incomplete reporting of follow-up. No randomized controlled trials were identified.

### Definitions of cervical and supraclavicular lymph node metastases

Substantial heterogeneity was observed in how CLNM and SCLNM were defined and classified across studies (Table 2). Terminology varied widely and included ‘cervical lymph node metastasis,’ ‘supraclavicular lymph node metastasis,’ ‘M1 lymph node metastasis,’ and ‘advanced nodal disease.’

**Table 2:** Definitions, diagnostic criteria, anatomical characterization, and extent of cervical lymph node metastases

Study, year	Terminology and staging			Diagnostic criteria	Anatomical characterization			Disease extent and burden			Assessment
	Terms used by authors	Staging label applied	Classification system reported	How were nodes defined	Anatomical nodal region described	Anatomical detail provided	Isolated cervical disease	Multi-station disease assessed	Nodal burden quantified	Laterality of CLNM reported	Timing of nodal assessment
Kato, 2000	CLNM	M1 LYM	TNM 4^th^ (1987)	Imaging + pathology	Cervical compartment	Regional	Yes (2/46)	Yes	No	NR	Baseline, pathological
Miyata, 2015	SCLN	Distant LN	UICC TNM 7^th^	CT ± PET	SCLN only	None	Unclear	Unclear	Yes	NR	Baseline, post-NAT
Liming, 2017	SCLN	Unclear	TNM based (not explicit)	CT + EUS ± biopsy	SCLN only	None	Unclear	Unclear	Yes	Yes	Baseline
Honma, 2017	SCLN	M1 LYM	UICC TNM 7^th^	CT + EUS ± PET	SCLN only	None	Unclear	Unclear	No	No	Baseline, pathological
Kang, 2017	CLNM	Advanced nodal disease	AJCC TNM 7^th^ + Head & Neck	Imaging + pathology	Cervical neck levels (I-IV)	Station-level	Unclear	Unclear	No	NR	Pathological
Chen, 2018	SCLN	Regional LN	AJCC TNM 7^th^	CT ± PET	SCLN (contour-based)	Regional	Unclear	Unclear	Yes	NR	Baseline
Chen, 2018	SCLN/Neck LN	Regional/Distant LN	AJCC TNM 7^th^	CT ± PET	SCLN vs other neck nodes	Regional	Unclear	Unclear	No	NR	Baseline
Chung, 2019	M1a	M1a vs regional	AJCC TNM 6^th^ – 8^th^	CT + PET + EUS	Supraclavicular	Regional	Unclear	Yes	No	NR	Baseline
Sato, 2021	SCLN	Stage IVB/regional	UICC TNM 8^th^; JES 11^th^	CT + PET + pathology	JES nodal stations #101, #104	Station-level	Yes, by inclusion	No	Yes	NR	Baseline, pathological
Pape, 2022	SCLN	Distant LN	AJCC TNM 7^th^	Registry imaging	SCLN only	None	Unclear	Unclear	No	NR	Baseline
Yu, 2022	SCLN	Distant LN	UICC TNM 7^th^	CT ± PET ± biopsy	SCLN only	None	Unclear	Unclear	Partially	Yes	Baseline, pathological
Horst, 2023	CLNM	Regional LN	AJCC TNM 8^th^ + Head & Neck	CT + PET + biopsy	Neck levels III-IV	Station-level	No	Yes	Yes	Yes	Baseline, pathological
Chang, 2023	SCLN	Distant LN	AJCC TNM 8^th^	Registry imaging	SCLN only	None	Unclear	Unclear	No	NR	Baseline
Igaue, 2024	M1LN	Resectable M1 LYM	JES 11^th^	CT ± PET + pathology	JES #104	Station-level	Unclear	Yes	Yes	NR	Baseline, pathological
Park, 2024	SCLN	Unclear	TNM based (not explicit)	CT + PET/pathology	SCLN only	None	Unclear	Indirect	Yes	NR	Baseline and pathological
Li, 2024	CLNM vs SCLNM	Regional/distant LN	JES vs AJCC	Unclear	CLN vs SCLN separated	Regional	Unclear	Unclear	No	NR	Unclear
Shiraishi, 2024	SCLN	Regional/distant LN	JES 11^th^	CT ± PET ± pathology	JES #101 vs #104	Station-level	Unclear	Unclear	Yes	NR	Baseline, pathological
Zeng, 2024	SCLN	Distant LN	AJCC + JES	CT ± PET	JES #104 vs non-#104	Station-level/regional	Unclear	Unclear	No	NR	Baseline
Zeng, 2025	Non regional CLNM	Non-regional	AJCC/UICC 8^th^ + JES	US + FNA + imaging	Neck levels reported IV-VI	Station-level	Yes, by inclusion	Yes	Yes	Yes	Baseline, pathological

AJCC, American Joint Committee on Cancer; CLNM, cervical lymph node metastases; CT, computed tomography; EUS, endoscopic ultrasound; FNA, fine-needle aspiration; JES, Japanese Esophageal Society; LN, lymph node; NR, not reported; PET, positron emission tomography; SCLN, supraclavicular lymph nodes; UICC, Union for International Cancer Control; US, ultrasound.

**Fig. 2:**
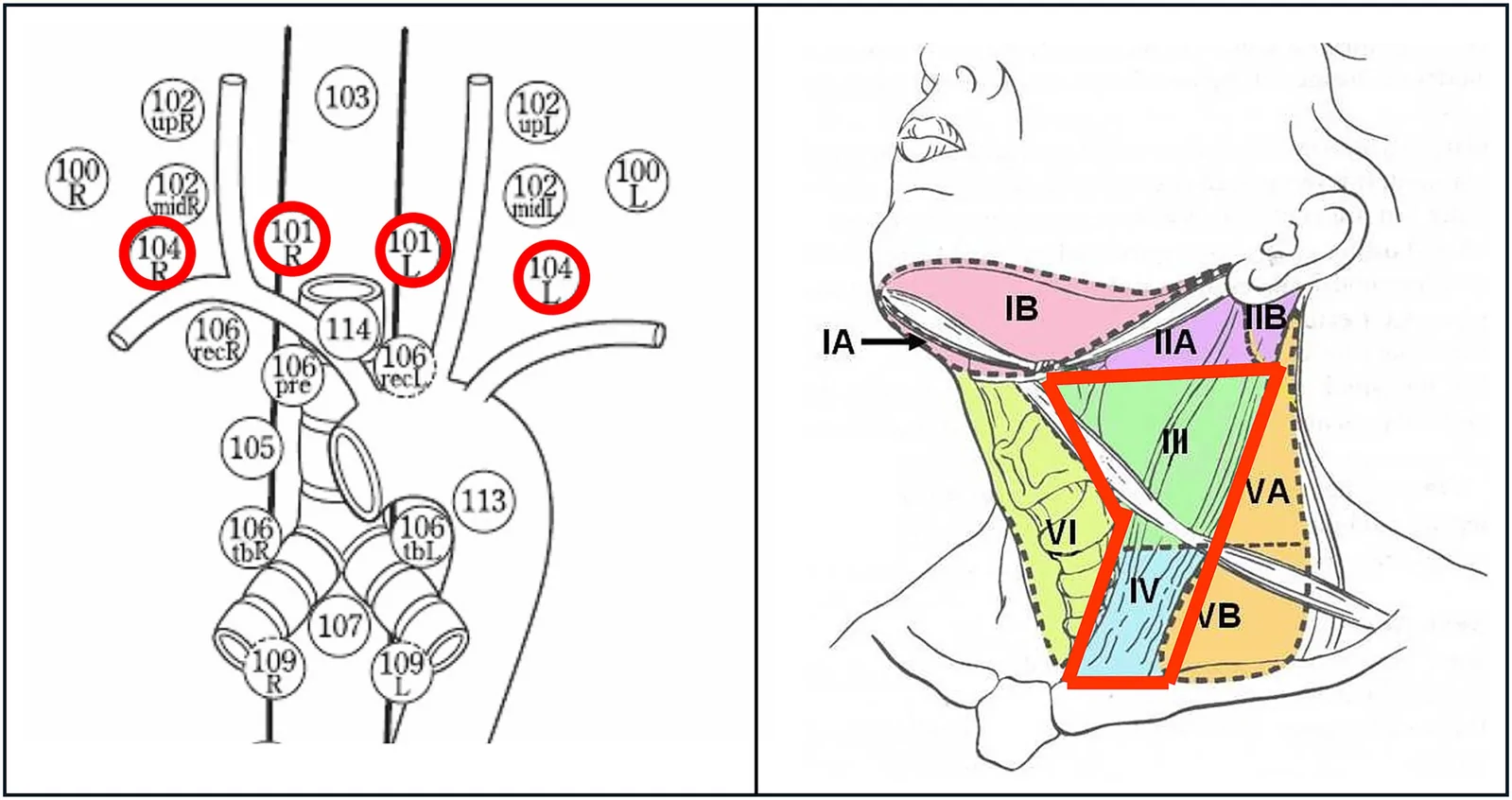
Anatomical classification of cervical nodal involvement in thoracic esophageal cancer. Left panel: Japanese Esophageal Society (JES) nodal station classification with highlighted cervical paraesophageal nodes (#101) and supraclavicular nodes (#104). Right panel: Cervical lymph node levels I–VI according to the American Head and Neck classification system, with levels III–IV highlighted. Partial anatomical overlap exists between JES cervical station #101 and head and neck levels III–IV; however, these systems are not equivalent and differ in anatomical boundaries, orientation, and surgical context. JES station #104 (supraclavicular nodes) also demonstrates partial correspondence with lower cervical levels, including level V, but does not align directly with head and neck level definitions. Left panel adapted from Mine S, Tanaka K, Kawachi H, *et al.* Japanese Classification of Esophageal Cancer, 12th Edition: Part I. Esophagus. 2024;21(3):179–215. Licensed under Creative Commons Attribution 4.0. Right panel adapted from Harish K. Neck dissections: radical to conservative. World J Surg Oncol. 2005;3:21. DOI: 10.1186/1477-7819-3-21. Licensed under Creative Commons Attribution 2.0.

Staging labels differed according to the classification system applied. Several studies classified cervical or supraclavicular lymph nodes as distant metastases using earlier or contemporary AJCC/UICC TNM editions,^11^^,^^14^^,^^21^^,^^22^^,^^24^ whereas others considered these nodes regional based on JES classification.^20^^,^^25^^,^^28^ Some studies explicitly acknowledged discrepancies between staging systems,^19^^,^^27–29^ while others applied staging labels without detailed justification.^15^^,^^16^^,^^26^ In a minority of studies, cervical nodal disease was analyzed without explicit staging categorization.^18^^,^^30^ The anatomical relationship between these classification systems is illustrated in Fig. 2.

### Anatomical characterization of cervical nodal disease

The level of anatomical detail used to define cervical nodal involvement differed markedly between studies (Table 2). In most reports, cervical or supraclavicular lymph nodes were treated as a single anatomical region without further subdivision. These studies relied primarily on imaging-based definitions, typically using computed tomography with or without positron emission tomography, and did not distinguish between specific cervical nodal stations or neck levels.

A minority of studies reported cervical nodal involvement at station- or neck-level resolution. Several surgical series incorporating three-field lymphadenectomy reported station-level involvement according to JES nodal definitions, most commonly distinguishing cervical paraesophageal nodes (#101) from supraclavicular nodes (#104).^20^^,^^25^^,^^28^^,^^29^ Other studies applied neck level–based classifications derived from head and neck oncology (e.g. levels I–IV or III–IV),^16^^,^^23^^,^^30^ while some distinguished supraclavicular from other cervical nodal regions without further anatomical specification.

Overall, fewer than half of the studies included allowed differentiation between distinct cervical nodal stations, and only a minority reported station-specific outcomes.

Beyond anatomical classification, reporting of disease extent and nodal burden was inconsistent (Table 2). Only two studies explicitly identified a subgroup of patients with isolated cervical nodal disease in the absence of additional mediastinal or abdominal lymph node involvement.^13^^,^^20^ In most studies, it remained unclear whether cervical nodal involvement occurred in isolation or as part of more extensive multistation nodal dissemination.

Quantification of nodal burden was reported in a subset of studies using heterogeneous metrics, including absolute lymph node counts, AJCC N stage, or the number of involved nodal regions. Laterality of cervical nodal involvement was infrequently documented, and compartment-specific nodal burden was rarely reported. Consequently, stratification of outcomes according to cervical-only versus multistation disease was not feasible in the majority of included studies.

### Treatment strategies and survival outcomes

Treatment strategies varied widely across studies and included multimodality approaches incorporating surgery, definitive chemoradiotherapy, and palliative treatments (Table 3). Surgical series most commonly employed neoadjuvant chemotherapy or chemoradiotherapy followed by esophagectomy with three-field lymphadenectomy, whereas non-surgical cohorts primarily received definitive chemoradiotherapy.

**Table 3:** Treatment and survival

Study, year	Treatment			Tumor characteristics		Overall Survival			
	Treatment (subgroup)	N	Intent	Histology	Anatomical nodal classification	Median (m)	1-yr	3-yr	5-yr
Kato, 2000	NAT + E + 3FL	46	Curative	SCC >95%	Cervical compartment	-	-	26%	16%
Miyata, 2015	NCT+ E + 3FL (Baseline SCLN+)	57	Curative	SCC 100%	SCLN only	-	-	40%	-
	NCT+ E + 3FL (post-NAT SCLN+)	47	Curative			-	-	20%	16%
Liming, 2017	IMRT + SCLN hyperthermia ± CT	50	Definitive	SCC 100%	SCLN only	29	83%	43%	-
Honma, 2017	nC(R)T + E + 3FL	45	Curative	SCC 100%	SCLN only	28	89%	43%	26%
	E + 3FL	19	Curative			51	79%	47%	41%
	dCRT	38	Curative			22	73%	21%	18%
Kang, 2017	NAT + E + 3FL	34	Curative	SCC 100%	Neck levels I-IV	-	-	-	23%
Chen, 2018	dCRT (SCLN)	70	Definitive	SCC 100%	SCLN contour-based	17	-	-	-
	dCRT (neck LN)	35	Definitive			10	-	-	-
Chung, 2019	dCRT	31	Definitive	SCC 100%	Supraclavicular	16	-	-	11%
Sato, 2021	nCRT + E + 3FL	18	Curative	SCC 100%	JES #101, #104	-	-	-	41%
Pape, 2022	CRT ± E	48	Unclear	SCC 100%	SCLN only	16	-	-	-
	Systemic therapy	15	Unclear			-	-	-	-
	RT/Stent	48	Palliative			6	-	-	-
Yu, 2022	NCT + E + 3FL	41	Curative	SCC 100%	SCLN only	N/A*	-	72%	-
	CRT	133	Definitive			20	-	36%	-
	RT	23	Palliative			11	-	19%	-
Horst, 2023	nCRT + E + 3FL	20	Curative	SCC 35%/AC 65%	Neck levels III-IV	-	85%	-	-
Chang, 2023	nCRT + E + 3FL	23	Curative	SCC 19%/AC 73%	SCLN only	39	-	51%	-
	E + 3FL	18	Curative			12	-	9%	-
	dCRT	51	Definitive			8	-	12%	-
Igaue, 2024	NAT + E + extended LND	80	NR	SCC 90%	JES #104	-	-	77%	-
Park, 2024	E + 3FL (clinically+)	149	Curative	SCC 100%	SCLN only	-	-	-	30%
	E + 3FL (pathologically+)	75	Curative			-	-	-	26%
Li, 2024	E + 3FL (CLNM)	52	Curative	SCC 100%	CLN	20	-	29%	21%
	E + 3FL (SCLNM)	64	Curative		SCLN	35	-	48%	34%
Shiraishi, 2024	NAT + E + 3FL (#101+)	NR	Curative	SCC 93%/AC 5%	JES #101	-	-	-	29%
	NAT + E + 3FL (#104+)	66	Curative		JES #104	-	-	-	32%
Zeng, 2024	dCRT (#104+ SCLN)	NR	Unclear	SCC 100%	JES #104	17	72%	11%	-
	dCRT (Non #104 CLNM)	NR	Unclear		JES non #104	28	81%	39%	-
Zeng, 2025	nCIT + E + 3FL	15	Curative	SCC 100%	Neck levels IV-VI	-	92% DFS†	-	-

AC, adenocarcinoma; 3FL, three-field lymphadenectomy; CLNM, cervical lymph node metastases; CRT, chemoradiotherapy; dCRT, definitive chemoradiotherapy; DFS, disease-free survival; E, esophagectomy; IMRT, intensity-modulated radiotherapy; JES, Japanese Esophageal Society; LN, lymph node; LND, lymph node dissection; m, months; NAT, neoadjuvant therapy; nC(R)T, neoadjuvant chemotherapy or chemoradiotherapy; NCT, neoadjuvant chemotherapy; nCIT, neoadjuvant chemo-immunotherapy; nCRT, neoadjuvant chemoradiotherapy; NR, not reported; RT, radiotherapy; SCC, squamous cell carcinoma; SCLN, supraclavicular lymph nodes; Stent, esophageal stenting; yr, year.

^*^N/A: Median OS not reached.

^†^DFS: only 1-yr DFS reported.

Survival outcomes differed substantially across treatment contexts and study populations. In surgical series, reported median overall survival ranged from approximately 20 to over 50 months, with 5-year overall survival rates generally between 20% and 40% in selected cohorts. Several surgical studies reported long-term survival in patients with cervical or supraclavicular nodal involvement, including series that distinguished outcomes by nodal location or station.

In contrast, studies evaluating definitive chemoradiotherapy reported shorter survival, with median overall survival typically ranging from 10 to 30 months. Within these cohorts, survival differed according to anatomical nodal location in some studies, with poorer outcomes observed in patients with supraclavicular or specific cervical nodal involvement compared with other cervical nodal regions.^17^^,^^29^

Registry-based and heterogeneous cohorts demonstrated broad survival ranges, reflecting variation in patient selection, histology, treatment allocation, and staging definitions. Across all treatment contexts, substantial heterogeneity in outcomes was observed, and direct comparison between strategies was limited by non-randomized study designs and selection bias.

Treatment-related morbidity was reported inconsistently across studies and was primarily described in surgical series. Postoperative complications were common, with recurrent laryngeal nerve palsy reported in approximately 20%–47% of patients undergoing three-field lymphadenectomy.^16^^,^^20^^,^^23^^,^^30^ Anastomotic leakage and pulmonary complications were also frequently observed. Reporting of treatment-related toxicity following definitive chemoradiotherapy was more limited and heterogeneous, but commonly included hematological and gastrointestinal adverse events. Detailed complication data are summarized in Supplementary Table S6.

## DISCUSSION

Cervical lymph node metastases in thoracic esophageal cancer, as reported in the existing literature, represent a heterogeneous and anatomically diverse clinical category. This review demonstrates marked variation in terminology, staging classification, anatomical characterization, and treatment strategies, reflecting the different perspectives on whether these metastases should be considered regional (N) or distant (M) disease. Such variability complicates interpretation of survival outcomes and limits meaningful comparison across studies.

Within this heterogeneous reporting, however, important anatomical distinctions emerge. In particular, involvement of lymph nodes along the longitudinal paraesophageal and parajugular chain, corresponding in head and neck classification systems to cervical levels III–IV, differs from more lateral or extensive cervical nodal dissemination (level II and V). Spread to these longitudinal chain lymph nodes may represent contiguous locoregional extension rather than systemic metastatic spread.^4^^,^^31^

Importantly, several surgical series report durable survival in selected patients treated with curative intent, including three-field lymphadenectomy, with five-year survival rates of approximately 20%–40%.^11^^,^^20^^,^^26–28^ Outcomes were most favorable in cohorts with limited disease burden and stringent selection.^22–24^ Together, these findings suggest that cervical nodal involvement confined to the longitudinal drainage pathway may be compatible with long-term disease control and outcomes more consistent with advanced regional nodal disease than with widespread metastatic dissemination.

In most studies, cervical or supraclavicular lymph nodes were treated as a single anatomical category without further subdivision. Only a minority provided station- or neck-level detail distinguishing longitudinal paraesophageal or parajugular chain nodes from more lateral cervical regions. As a result, patients with longitudinal chain involvement, potentially compatible with locoregional (N) disease, were frequently analyzed together with those exhibiting more extensive or anatomically distinct cervical dissemination. This limited anatomical stratification likely obscured clinically relevant differences in disease behavior, as reported survival outcomes reflect a composite of distinct patterns of spread rather than a uniform clinical entity.

Beyond nodal location, the overall distribution and burden of lymphatic spread were infrequently reported. Few studies distinguished cervical involvement confined to the upper mediastinum and neck from multistation thoracic or abdominal dissemination.^13^^,^^20^ Laterality was rarely documented; in the limited series reporting side-specific involvement, residual cervical metastases were predominantly ipsilateral to the preoperatively identified nodes, with unexpected contralateral involvement observed only in a small minority of patients.^23^ Where nodal burden or extent was examined, more extensive disease was generally associated with poorer outcomes, indicating that distribution and burden may be at least as influential as anatomical location itself.^22^^,^^26^^,^^27^

Diagnostic heterogeneity further limits interpretation. Only 10 of 19 cohorts confirmed cervical nodal involvement pathologically, while the remainder relied primarily on imaging-based assessment, introducing potential misclassification (Table 2). The implications of imaging-based nodal assessment for survival interpretation warrant careful consideration. False-positive cervical nodal identification on computed tomography or positron emission tomography is well recognized, particularly in the setting of reactive lymphadenopathy or inflammatory changes.^32^ Inclusion of patients without true nodal metastasis in imaging-defined cohorts may artificially inflate survival estimates, as these patients would effectively represent a lower disease burden than classified. Conversely, understaging due to limited sensitivity for small-volume nodal disease may also occur. In either direction, misclassification introduces systematic error into survival analyses, complicating interpretation of both prognosis and treatment effect. This limitation is particularly consequential in the context of the present review, where anatomical location and extent of nodal involvement form the basis of the central argument. Conclusions regarding the locoregional versus metastatic behavior of cervical nodal disease are inherently dependent on the accuracy of nodal staging and should be interpreted with appropriate caution in cohorts relying exclusively on imaging-based assessment.

Notwithstanding these diagnostic limitations, the available evidence supports anatomically precise stratification of cervical nodal disease as a meaningful step toward improved clinical interpretation. In Western practice, where cervical nodes are often categorized as distant metastases, our findings support reconsideration of nodal involvement confined to the longitudinal paraesophageal and parajugular chain (corresponding to head and neck levels III–IV) within a locoregional framework, particularly when occurring as part of a contiguous lymphatic spread rather than extensive multicompartment nodal dissemination. Clear differentiation between longitudinal chain involvement and more widespread cervical or multistation disease may improve treatment selection and clarify prognostic interpretation.

The evolution of esophageal cancer staging in relation to celiac axis lymph nodes provides an instructive precedent. Celiac nodes were historically classified as distant (M1a) disease, but were later redefined as regional nodal involvement following improved anatomical understanding and survival data.^9^^,^^19^ This historical shift illustrates that rigid locoregional–metastatic distinctions may require reassessment when anatomical and outcome data suggest a more nuanced pattern of spread.

Interpretation of the available evidence is further shaped by geographic and histological distribution. Most included studies originated from East Asia and consisted predominantly of SCC cohorts, reflecting regional epidemiology and surgical practice patterns.^3^ In these settings, cervical nodal involvement is more often approached within a regional framework depending on primary tumor location, frequently incorporating three-field lymphadenectomy.^12^^,^^33^^,^^34^ In contrast, Western cohorts, where AC predominates, have traditionally classified cervical nodes as distant metastases, favoring non-surgical or definitive chemoradiotherapy strategies.^23^ These differing staging philosophies and histological distributions result in fundamentally different patient populations and treatment intents being represented under similar terminology.

Moreover, AC remains underrepresented in the available literature, limiting histology-specific conclusions and underscoring the need for dedicated studies in this subgroup. Recent population-based data from Western cohorts may begin to address this gap. A Dutch nationwide cohort study, published after the search cutoff of this review, included both SCC and AC patients with thoracic esophageal cancer and concurrent cervical nodal metastasis, providing relevant context for Western applicability.^35^ These findings further highlight the diagnostic importance of pathological confirmation of nodal disease. Neoadjuvant therapy followed by surgery was associated with longer survival compared with definitive chemoradiotherapy, though baseline differences and residual confounding limit causal interpretation. Survival outcomes were broadly comparable between histological subtypes within each treatment group, and histology was not independently associated with survival in multivariable analysis, suggesting that outcome differences between SCC and AC may reflect treatment selection rather than intrinsic biological behavior. Dedicated prospective studies in Western, AC-dominant cohorts are needed to clarify whether the anatomical and prognostic patterns described in this review translate across histological subtypes.

This review has several strengths. It represents the first systematic synthesis focused specifically on CLNM in thoracic esophageal cancer. By integrating data across staging systems, geographic regions, and treatment strategies, and by evaluating anatomical definitions and reporting practices in addition to survival outcomes, it provides a structured framework for interpreting this heterogeneous literature.

Several limitations should be acknowledged. The available evidence is derived almost exclusively from retrospective, predominantly single-center cohort studies with inherent risk of selection bias and confounding by indication. Considerable variability existed in anatomical definitions, staging classification, diagnostic confirmation, and reporting of disease extent, limiting cross-study comparability and precluding quantitative synthesis. Importantly, this heterogeneity reflects not only a limitation of the present review but also insufficient standardization in reporting practices across the existing literature. The absence of consensus definitions for cervical nodal stations, inconsistent application of staging systems, variable documentation of disease extent and nodal burden, and underrepresentation of AC represent fundamental gaps in the field that limit the interpretability of individual studies and collective evidence alike.

Future research should prioritize anatomically precise reporting of cervical nodal involvement, including explicit documentation of nodal stations, disease distribution, and burden when evaluating treatment effects and outcomes. Prospective, multicenter collaboration across differing staging frameworks may further clarify the clinical significance of longitudinal cervical spread.

## CONCLUSION

Cervical lymph node metastases in thoracic esophageal cancer should not be regarded as a uniform disease entity. Available evidence indicates that involvement confined to the longitudinal paraesophageal and parajugular drainage pathway may behave more similarly to locoregional (N) disease than to unequivocal M1 systemic metastatic spread. In selected patients with anatomically confined cervical involvement treated with curative intent, long-term survival has been reported. Improved anatomical stratification and consistent reporting are essential to clarify prognosis and guide treatment decisions. These findings support a more individualized approach to staging: patients with longitudinal chain involvement should not be categorically excluded from curative-intent treatment, and three-field lymphadenectomy warrants consideration in carefully selected cases within experienced multidisciplinary teams.

## Conflicts of interest

J.P. Ruurda has served as a proctor for Intuitive Surgical and as a consultant for Medtronic. R. van Hillegersberg has served as a proctor for Intuitive Surgical, a consultant for Medtronic, and a member of the advisory board for Olympus. N. Haj Mohammad declares consultancy roles for AstraZeneca, Bristol Myers Squibb, Merck, and Servier, all paid to her institution. The remaining authors declare no competing interests."

## Supplementary Material

DOTE_Cervical_LNM_Supplementary_Tables_1_6_doag068

## Data Availability

All data included in this review were extracted from previously published studies and are presented within the manuscript and its supplementary materials. No new datasets were generated.

## Notes

FE Lammes and S Kalash contributed equally.

Specific author contributions: Maxime Eduard Sanders (Conceptualization, Data curation, Formal analysis, Investigation, Methodology, Project administration, Resources, Software, Supervision, Validation, Visualization, Writing—original draft), Femke Lammes (Conceptualization, Data curation, Formal analysis, Methodology, Writing—original draft), Sam Kalash (Conceptualization, Data curation, Formal analysis, Methodology, Writing—original draft), Sylvia van der Horst (Conceptualization, Methodology, Supervision, Writing—original draft), Stella Mook (Conceptualization, Methodology, Supervision, Validation, Writing—original draft), Teus Weijs (Data curation, Investigation, Methodology, Supervision, Writing—original draft), Jan Erik Freund (Formal analysis, Methodology, Supervision, Writing—original draft), Bas L Weusten (Formal analysis, Methodology, Supervision, Writing—original draft), Gert Meijer (Formal analysis, Methodology, Supervision, Writing—original draft), Nadia Haj Mohammad (Formal analysis, Methodology, Supervision, Writing—original draft), Adam Zeyara (Formal analysis, Methodology, Supervision, Writing—original draft), Jelle Ruurda (Conceptualization, Formal analysis, Investigation, Methodology, Project administration, Supervision, Writing—original draft), and Richard van Hillegersberg (Conceptualization, Data curation, Formal analysis, Investigation, Methodology, Project administration, Supervision, Validation, Visualization, Writing—original draft).

Financial support: None.

Potential competing interests: None.

Funding: This research received no specific grant from any funding agency in the public, commercial, or not-for-profit sectors.
