# Supplementary material for: Cervical lymph node metastases in thoracic esophageal cancer: a systematic review
Source: Dis Esophagus. 2026 Jul 6;39(4):doag068. doi: 10.1093/dote/doag068 (PMC13336634; doi:10.1093/dote/doag068)
Supplement: DOTE_Cervical_LNM_Supplementary_Tables_1_6_doag068 [file dote_cervical_lnm_supplementary_tables_1_6_doag068.docx]

**Supplementary Table S1:** ***Search string Pubmed.***

*Last updated on 20th of October 2025.*

|  |  | Pubmed | |
| --- | --- | --- | --- |
|  |  | Search | Results |
| Patient | A | "esophagus cancer"[Title/Abstract] OR "esophageal cancer"[Title/Abstract] OR "cancer of the esophagus"[Title/Abstract] OR "oesophageal cancer"[Title/Abstract] OR "oesophagus cancer"[Title/Abstract] OR "esophageal carcinoma"[Title/Abstract] OR "carcinoma of the esophagus"[Title/Abstract] OR "cancer of the oesophagus"[Title/Abstract] OR "esophageal squamous cell carcinoma"[Title/Abstract] OR "oesophageal squamous cell carcinoma"[Title/Abstract] OR "esophageal squamous carcinoma"[Title/Abstract] OR "oesophageal squamous carcinoma"[Title/Abstract] OR "esophageal adenocarcinoma"[Title/Abstract] OR "oesophageal adenocarcinoma"[Title/Abstract] OR "gastroesophageal cancer"[Title/Abstract] OR "gastroesophageal carcinoma"[Title/Abstract] OR "esophagogastric cancer"[Title/Abstract] OR "oesophagogastric cancer"[Title/Abstract] OR "esophagogastric carcinoma"[Title/Abstract] OR "oesophagogastric carcinoma"[Title/Abstract] | 57,964 |
|  | B | "cervical lymph node metastas*"[Title/Abstract] OR "cervical lymph metastas*"[Title/Abstract] OR "neck lymph node metastas*"[Title/Abstract] OR "neck lymph metastas*"[Title/Abstract] OR "supraclavicular lymph node metastas*"[Title/Abstract] OR "distant lymph node metastas*"[Title/Abstract] OR "extraregional lymph node metastas*"[Title/Abstract] OR "extra regional lymph node metastas*"[Title/Abstract] OR "m1a node involvement"[Title/Abstract] OR "m1 lymph node metastas*"[Title/Abstract] | 4,409 |
|  | **A+B** | **Combined (Patients with esophageal cancer + cervical lymph node metastases)** | **216** |

**Supplementary Table S2*: Search string EMBASE.***

*Last updated on 20th of October 2025.*

|  |  | Embase | |
| --- | --- | --- | --- |
|  |  | Search | Results |
| Patient | A | 'esophagus cancer':ab,ti OR 'esophageal cancer':ab,ti OR 'cancer of the esophagus':ab,ti OR 'oesophageal cancer':ab,ti OR 'oesophagus cancer':ab,ti OR 'esophageal carcinoma':ab,ti OR 'carcinoma of the esophagus':ab,ti OR 'cancer of the oesophagus':ab,ti OR 'esophageal squamous cell carcinoma':ab,ti OR 'oesophageal squamous cell carcinoma':ab,ti OR 'esophageal squamous carcinoma':ab,ti OR 'oesophageal squamous carcinoma':ab,ti OR 'esophageal adenocarcinoma':ab,ti OR 'oesophageal adenocarcinoma':ab,ti OR 'gastroesophageal cancer':ab,ti OR 'gastroesophageal carcinoma':ab,ti OR 'esophagogastric cancer':ab,ti OR 'oesophagogastric cancer':ab,ti OR 'esophagogastric carcinoma':ab,ti OR 'oesophagogastric carcinoma':ab,ti | 83,839 |
|  | B | 'supra-clavicular lymph node metastas*':ab,ti OR 'supraclavicular lymph node metastas*':ab,ti OR 'supraclavicular lymph metastas*':ab,ti OR 'cervical lymph node metastas*':ab,ti OR 'cervical lymph metastas*':ab,ti OR 'neck lymph node metastas*':ab,ti OR 'neck lymph metastas*':ab,ti OR 'distant lymph node metastas*':ab,ti OR 'extra-regional lymph node metastas*':ab,ti OR 'extraregional lymph node metastas*':ab,ti OR 'm1 lymph node metastas*':ab,ti | 5,651 |
|  | **A+B** | **Combined (Patients with esophageal cancer + cervical lymph node metastases)** | **308** |

|  |  | Cochrane | |
| --- | --- | --- | --- |
|  |  | Search | Results |
| Patient | A | (esophageal NEXT (cancer or carcinoma)):ti,ab,kw OR (oesophageal NEXT (cancer or carcinoma)):ti,ab,kw OR (esophageal NEXT (squamous cell carcinoma)):ti,ab,kw OR (esophageal NEXT (adenocarcinoma)):ti,ab,kw OR (gastroesophageal NEXT (cancer or carcinoma)):ti,ab,kw | 5,401 |
|  | B | (cervical NEXT (lymph node metastas*)):ti,ab,kw OR (neck NEXT (lymph node metastas*)):ti,ab,kw OR (supraclavicular NEXT (lymph node metastas*)):ti,ab,kw OR (extraregional NEXT (lymph node metastas*)):ti,ab,kw | 821 |
|  | **A+B** | **Combined (Patients with esophageal cancer + cervical lymph node metastases)** | **58** |

**Supplementary Table S3*: Search string Cochrane.***

*Last updated on 20th of October 2025.*

**Supplementary Table S4. Newcastle–Ottawa Scale scoring criteria applied in this review**

| **Domain** | **NOS item** | **Criterion for awarding a point in this review** |
| --- | --- | --- |
| **Selection** | Representativeness of the exposed cohort | Point awarded if cohort was consecutive, population-based, or registry-derived |
|  | Selection of the non-exposed cohort | Point awarded only if an internal comparison group without CLNM/SCLNM was explicitly defined |
|  | Ascertainment of exposure | Point awarded if CLNM/SCLNM was confirmed by pathology, biopsy, or clearly defined imaging criteria |
|  | Outcome not present at start | Point awarded if baseline absence of outcome (death) was explicitly stated or implicit by design |
| **Comparability** | Control for confounding (1) | Point awarded if analysis adjusted for ≥1 key confounder (e.g. T stage, N stage, age) |
|  | Control for confounding (2) | Second point awarded if multivariable analysis or propensity score methods were used |
| **Outcome** | Outcome assessment | Point awarded if survival outcomes were ascertained via medical records, registry linkage, or standardized follow-up |
|  | Adequacy of follow-up duration | Point awarded if median follow-up ≥12 months or 3-year survival reported |
|  | Adequacy of follow-up completeness | Point awarded if follow-up was complete or loss to follow-up <20%, or clearly reported |

**Supplementary Table S5. Iten-level Newcastle-Ottawa Scale scoring of included studies**

| **Studies** | **Selection** | | | | **Comparability** | **Outcome** | | | **Total score** | **Risk category** |
| --- | --- | --- | --- | --- | --- | --- | --- | --- | --- | --- |
|  | Exposed truly representative of average | Selection of non-exposed from the same community | Exposure ascertained by secure records or interview | Demonstration of outcome of interest not present at the start of the study | Study controls for other variables (sex, age, staging) | Assessment of outcome by independent blind assessment or record linkage | Follow-up long enough for outcome to occur | Complete follow-up for all subjects accounted for |  |  |
| Kato, 2000 | **x** | **x** | **✓** | **✓** | **x x** | **✓** | **✓** | **✓** | 4 | Higher |
| Miyata, 2015 | **x** | **x** | **✓** | **✓** | **✓ x** | **✓** | **✓** | **x** | 5 | Moderate |
| Liming, 2017 | **x** | **x** | **✓** | **✓** | **✓ x** | **✓** | **✓** | **x** | 5 | Moderate |
| Honma, 2017 | **x** | **x** | **✓** | **✓** | **✓ x** | **✓** | **✓** | **x** | 5 | Moderate |
| Kang, 2017 | **x** | **x** | **✓** | **✓** | **✓ x** | **✓** | **✓** | **x** | 5 | Moderate |
| Chen, 2018 | **✓** | **x** | **✓** | **✓** | **✓ x** | **✓** | **✓** | **x** | 6 | Moderate |
| Chen, 2018 | **✓** | **x** | **✓** | **✓** | **✓ x** | **✓** | **✓** | **x** | 6 | Moderate |
| Chung, 2019 | **✓** | **x** | **✓** | **✓** | **✓ x** | **✓** | **✓** | **x** | 6 | Moderate |
| Sato, 2021 | **x** | **x** | **✓** | **✓** | **✓ x** | **✓** | **✓** | **x** | 5 | Moderate |
| Pape, 2022 | **✓** | **x** | **x** | **✓** | **✓ x** | **✓** | **✓** | **x** | 6 | Moderate |
| Yu, 2022 | **✓** | **x** | **✓** | **✓** | **✓ ✓** | **✓** | **✓** | **x** | 7 | Lower |
| Horst, 2023 | **✓** | **x** | **✓** | **✓** | **✓ x** | **✓** | **x** | **✓** | 6 | Moderate |
| Chang, 2023 | **✓** | **x** | **x** | **✓** | **✓ ✓** | **✓** | **✓** | **✓** | 7 | Lower |
| Igaue, 2024 | **✓** | **x** | **✓** | **✓** | **✓ ✓** | **✓** | **✓** | **x** | 7 | Lower |
| Park, 2024 | **✓** | **x** | **✓** | **✓** | **✓ x** | **✓** | **✓** | **x** | 6 | Moderate |
| Li, 2024 | **x** | **x** | **x** | **✓** | **✓ x** | **✓** | **✓** | **x** | 4 | Higher |
| Shiraishi, 2024 | **x** | **x** | **✓** | **✓** | **✓ x** | **✓** | **✓** | **x** | 5 | Moderate |
| Zeng, 2024 | **x** | **x** | **✓** | **✓** | **✓ x** | **✓** | **✓** | **x** | 5 | Moderate |
| Zeng, 2025 | **x** | **x** | **✓** | **✓** | **x x** | **✓** | **x** | **✓** | 4 | Higher |

**Supplementary Table S6: Recurrence patterns and treatment-related complications reported in included studies of cervical or supraclavicular lymph node metastases**

| Study | Treatment | Recurrence | | | Complication | |
| --- | --- | --- | --- | --- | --- | --- |
|  |  | N (%) | 1-year | 5-year | Surgical | N (%) |
| Liming, 2017 | IMRT + HT + CT | 31 (62%) | NR | NR | NR | NR |
| Honma, 2017 | E + BCL + CT/RT  E + BCL  dCRT | 24 (53%)  9 (47.4%)  9 (23.7%) | NR | NR | Sepsis  Tracheal stenosis | NR |
| Kang, 2017 | NAT + E + 3FL | NR |  | 16.3% | RLN palsy  Anastomotic leakage  Pneumonia | 16 (47.1%)  1 (2.9%)  5 (14.7%) |
| Sato, 2021 | NCRT + E + 3FL | 8 (44.4%) | NR | NR | RLN palsy  Anastomotic leakage | 4 (22.2%)  2 (11.1%) |
| Van der Horst, 2023 | NCRT + E + 3FL | NR | 50% | NR | RLN palsy  Anastomotic leakage  Pneumonia  Chyle leakage  Pneumothorax  Pulmonary embolism | 9 (45%)  6 (30%)  8 (40%)  5 (25%)  2 (10%)  1 (5%) |
| Shiraishi, 2024 | NAT + E + 3FL | NR | NR | #101: 28.8%  #104: 32.3% | NR | NR |
| Zeng, 2025 | NCIT + E + 3FL | NR | NR | NR | Recurrent nerve injury  Anastomotic leakage  Atelectasis  Pulmonary dysfunction  Pneumonia  Pneumothorax | 3 (20%)  3 (20%)  7 (46.7%)  5 (33.3%)  5 (33.3%)  3 (20%) |

RFS: Recurrence-free survival; IMRT: Intensity-modulated radiotherapy; HT: Hyperthermia; CT: Chemotherapy; NR = Not reported; E = Esophagectomy; BCL = Bilateral cervical lymphadenectomy; RT = Radiotherapy; dCRT = Definitive chemoradiotherapy; NAT = Neoadjuvant therapy; 3F = 3-Field lymphadenectomy; NCRT = Neoadjuvant chemoradiotherapy; NCIT = Neoadjuvant chemoimmunotherapy; RLN: recurrent laryngeal nerve.
